# From knowledge to collective action: a peer-led model for climate and health leadership

**DOI:** 10.3389/fpubh.2026.1805050

**Published:** 2026-03-30

**Authors:** Jo Bjorgaard, Gloria E. Barrera

**Affiliations:** 1 Climate Advocacy Lab, Washington, DC, United States; 2 RISE Climate + Health Collective, Saint Paul, MN, United States; 3 School of Nursing, University of Minnesota Twin Cities, Minneapolis, MN, United States; 4 School of Nursing, University of Illinois Chicago School of Nursing, Chicago, IL, United States

**Keywords:** climate and health, climate communication, health professional education/courses, public health advocacy, workforce capacity building

## Abstract

The effects of climate change are increasingly impacting population health, yet most health professionals lack training in communication, advocacy, and systems-level leadership. This paper describes the Climate and Health Peer Learning Circle (PLC), a national, community-based leadership program developed by the Climate Advocacy Lab to strengthen health professional capacity for climate-responsive education and advocacy. Using a peer learning and practice-based model, the PLC supports participants to develop skills in narrative strategy, relational organizing, strategic communication, and applied advocacy. This manuscript presents the program’s conceptual framework, curriculum design, and implementation across four regional cohorts in 2025, and summarizes practice-based feedback gathered through surveys, coaching sessions, and participant reflection. While not a formal research evaluation, findings suggest that participants increased their confidence and applied skills and used PLC strategies to advance climate and health initiatives in clinical, community, and professional settings. The PLC demonstrates the potential of relational, peer informed models to move beyond knowledge acquisition and support health professionals as credible communicators and civic leaders within the climate and health movement.

## Introduction

Climate change is increasingly recognized as one of the greatest threats to health in the 21st century (1). Health professionals hold a uniquely powerful position in this crisis: they are direct witnesses to climate-related health harms and among the most trusted voices in their communities (2, 3). This dual role gives them a distinct opportunity to help the public understand climate-related health risks, mobilize communities, and advance climate-responsive solutions. However, most health professionals receive little formal training in climate communication, advocacy, or systems-level leadership (4–6).

As climate-related health threats intensify, this gap limits the ability of the healthcare workforce to act effectively as educators, organizers, and advocates for community and planetary health (7). Despite mounting evidence that climate change is increasing heat related illness, respiratory and cardiovascular disease, mental health distress, and population displacement, the workforce remains inadequately prepared to respond (1, 8, 9).

### Problem statement

Climate related exposures including extreme heat, wildfires, flooding, and air pollution are occurring with greater frequency and severity, directly affecting individuals and communities across the lifespan (1, 8, 9). Yet climate and environmental health competencies remain inconsistently integrated into health professional education and workforce development (6, 10, 11). This disconnect creates a critical gap between the health impacts communities are experiencing and the capacity of clinicians to prevent, recognize, and mitigate climate related health harms (6, 11, 12).

Across undergraduate, graduate, and continuing professional education, training in climate and health is still largely knowledge-based, with limited emphasis on applied skills such as public communication, community engagement, advocacy, or systems change (12, 13). As a result, health professionals are increasingly expected to serve as trusted messengers and community advocates without the competencies needed to navigate complex systems, build coalitions, or mobilize collective action (3, 4, 6).

Few published models describe how to prepare health professionals for these expanded leadership roles through community-based, peer-driven, and advocacy-centered practical training. This lack of practice-based evidence limits the field’s ability to scale climate-responsive health leadership.

## Context

This manuscript presents the PLC as a community-based leadership training model and provides an overview of its 2025 regional cohort rollout, highlighting emerging practice-based impacts across clinical, community, and policy settings. Rather than reporting formal research outcomes, it offers a replicable training model and actionable guidance for educators, health systems, and public health leaders seeking to expand strategic movement capacity within the climate-health field.

### Setting: the climate advocacy lab

The Climate Advocacy Lab (CAL) is a U. S. based membership organization that provides evidence-informed tools, training, and learning infrastructure to support climate advocacy (14). The PLC was developed within CAL to address the need for applied skills in climate communication, advocacy, and systems change among health professionals (15).

### Program overview: climate and health peer learning circle

The PLC is a national leadership training program that equips health professionals—health system executives, nurses, physicians, health educators, public health workers, and allied health professionals—with practical advocacy and communication skills grounded in climate justice (15). Using participatory peer learning, one-to-one coaching, and practice-based skill development, the PLC empowers participants to lead climate-responsive initiatives in their communities and workplaces.^1^

**Figure 1:**
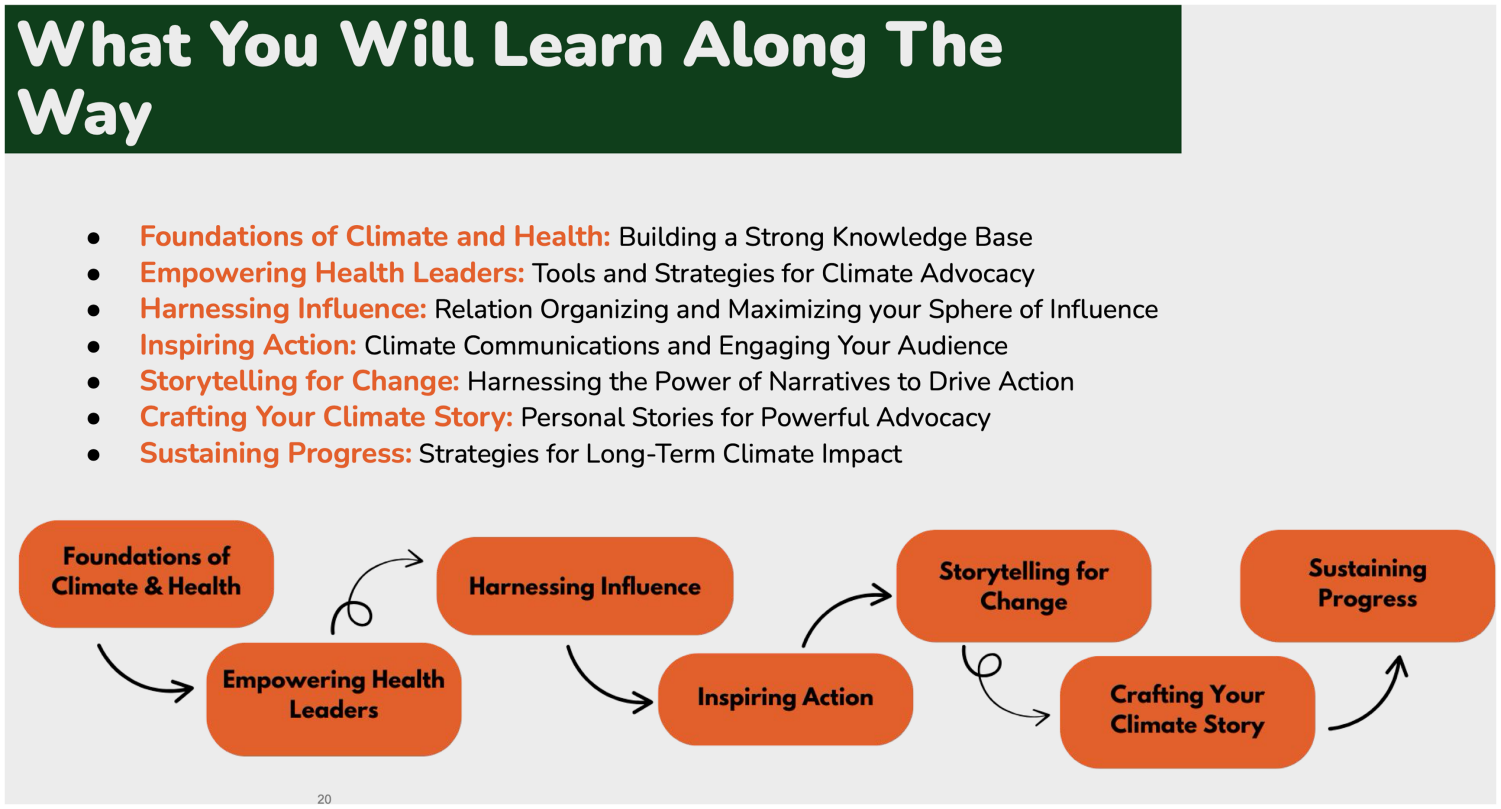
Climate and health PLC learning Arc.

Unlike many climate and health education and training programs that focus primarily on knowledge acquisition or clinical preparedness, the PLC is intentionally designed as a leadership and movement-building intervention. It integrates narrative strategy, relational organizing, strategic communication, and applied advocacy practice, positioning health professionals not only as educators, but as civic leaders and catalysts for structural change (Figure 1) (7, 10). Central to the model is the requirement that participants engage with an active project or campaign, ensuring that learning is immediately applied and results in tangible movement-building action throughout the training period.

### Participant recruitment

The PLC is open to interdisciplinary health professionals and health allies already working and leading at the intersection of climate and health. Participants were recruited for their respective regional cohorts through the CAL’s membership network, partner organizations, and health professional associations, and were required to apply during a designated application period. Eligibility criteria included being at least 18 years of age and based in the United States. Applications were reviewed and scored using predefined selection criteria, with the aim of building a cohort that reflected professional, geographic, and racial diversity and a range of advocacy experience.

### Conceptual framework

The Climate and Health PLC is informed by three complementary traditions that shape how learning, leadership, and action are cultivated: communities of practice, popular education, and contemporary social movement building. Rather than serving as abstract theory, these traditions act as organizing principles.

Drawing from communities of practice, the PLC is intentionally cohort-based and relationship-centered, emphasizing peer coaching, collaborative skill-building, and sustained connection through an alumni network (16–18). This structure supports continuous learning, reinforces professional identity as climate and health leaders, and fosters durable networks that extend beyond the formal training period.

The program is also grounded in popular education, which centers lived experience, dialog, and action as drivers of learning and social change (19–21). Participants enter the PLC with active projects rooted in their professional or community contexts. Learning is immediately applied, refined through peer feedback, and strengthened through structured reflection. In this model, participants are positioned practitioners and co-creators of knowledge rather than recipients of expert instruction.

Finally, the PLC is shaped by social movement and collective action approaches that emphasize narrative, relational organizing, and coordinated effort as mechanisms for structural change (22, 23). The program’s focus on storytelling, community advocacy, and cross-sector collaboration reflects the inherently social and political nature of climate and health work. By embedding these elements throughout the curriculum, the PLC supports participants in translating individual leadership development into collective, justice-oriented capacity.

## Detail to understand key programmatic elements

### Program development

Before the 2025 regional rollout, the Climate Advocacy Lab implemented two virtual PLC cohorts from 2022 to 2024—one for organizational representatives and one for individual health professionals. These six-month pilot programs trained 50 participants from leading climate and health organizations, including the Medical Society Consortium on Climate and Health, Healthcare Without Harm, EcoAmerica, and Physicians for Social Responsibility. The virtual model emphasized foundational competencies in advocacy, climate communication, relational organizing, and peer support.

Lessons from these pilots directly informed the development of the in-person, regionally tailored PLC model. They clarified which skills translated most effectively across professional roles, revealed structural barriers faced by practitioners, and underscored the value of collaborative, community-rooted learning environments.

The PLC is now offered as a month-long hybrid program that brings together interdisciplinary health professionals to strengthen skills in climate communication, organizing, narrative strategy, and evidence-based advocacy. Grounded in adult learning, application-driven approaches, and movement-building principles, the program prioritizes practical, immediately applicable competencies that support climate action across clinical, community, and policy settings (17, 20, 22). Program design also draws on social movement and collective action principles including storytelling to shape shared narratives and relational organizing to mobilize networks and build collective capacity for change. These approaches encourage participants to connect their professional roles to broader community and civic engagement strategies. The PLC curriculum therefore focuses on developing a set of core competencies, including strategic climate communication, narrative development, relational organizing, and applied advocacy planning, which participants apply directly to their ongoing projects and campaigns throughout the program (see Supplementary material).

Participants enter the PLC program already engaged in a variety of efforts aimed at addressing the health impacts of climate change in clinical, community, academic, and policy settings. In this program, the term “campaign” refers to a strategic set of activities, such as advocacy, public communication, research, education, or community engagement, designed to advance action on a particular climate and health issue. Some participants focus on integrating climate and health content into nursing or health professional education, while others work with local partners to strengthen climate resilience in neighborhoods facing risks such as flooding or extreme heat. Additional projects include initiatives to reduce industrial pollution affecting frontline communities, expand public engagement around climate and health through youth and faith-based programs, and develop creative approaches to climate literacy, including arts-based education efforts. Other participants are addressing upstream drivers of climate-related health risks through work on plastic pollution reduction, regenerative agriculture and food systems, or research exploring how access to urban greenspace may influence climate awareness and mental health among young people. Together, these initiatives illustrate how participants apply advocacy, communication, and organizing strategies to address climate-related health threats such as, extreme heat, poor air quality, food system instability, and mental health stressors, while supporting community resilience and advancing environmental justice.

The program brings together professionals from a wide range of backgrounds—including nurses, physicians, public health professionals, community health workers, environmental health practitioners, health communicators, and health system leaders–creating a multidisciplinary learning environment that reflects the complexity of climate-health challenges. Many work beyond traditional healthcare settings, serving in community-based organizations, advocacy groups, professional associations, and public health agencies. This diversity enriches dialog but also strengthens participants’ capacity to serve as credible messengers and effective advocates within their institutions and communities (24–26).

### Program delivery

The PLC curriculum is designed and primarily facilitated by a clinician with doctoral-level training in nursing practice and expertise in climate and health leadership development. Program delivery occurs in collaboration with Climate Advocacy Lab staff and invited subject-matter experts. For example, a consultant specializing in climate storytelling leads narrative and storytelling modules across cohorts, and regional climate and health leaders are occasionally invited to share local initiatives and strategies for engaging communities.

The program begins with a two-day regional, in-person workshop covering climate-health communication, narrative and message framing, audience engagement, advocacy fundamentals, structural inequities shaping climate and health risks, and grassroots community organizing (23, 27, 28). Instructional methods include brief lectures, facilitated discussion, small-group workshops, application exercises, and network building, using a train the trainer approach (29, 30). Participants apply for the cohort with an active project or campaign, allowing them to workshop and apply PLC learnings in real time.

Over the following month, participants complete asynchronous modules, skills-application assignments, and facilitated virtual sessions, and collaborate through structured exercises and peer feedback. Each participant also completes at least one peer coaching session, typically conducted virtually and lasting approximately 45–60 min. Coaching partners are fellow PLC participants who have completed the in-person training and are working on active climate and health initiatives within their own community contexts. Sessions follow a structured peer-coaching format that includes goal clarification, message or strategy review, and collaborative problem solving, while remaining flexible to address the participants specific campaign needs. Coaching conversations focus on refining messages, developing advocacy plans, identifying strategic audiences, and addressing barriers to climate-health engagement. Peer coaching supports accountability, shared leadership, and psychological safety (30, 31).

After the intensive, participants join a national community of practice offering virtual convenings, campaign workshops, resource development, cross-region networking, and opportunities for joint advocacy. This network supports ongoing learning and long-term leadership development and contributes to a growing pipeline of climate and health leaders (18, 32, 33).

### Program curriculum

In-person modules focus on foundational climate and health concepts, campaign development, community organizing, strategic communication, and storytelling, including the development of personal and community narratives. Virtual modules build on this content by emphasizing strategic planning, digital advocacy, coalition building, and sustaining long-term engagement. A complete module sequence and program framework is provided in Table 1. Participants also receive access to a digital resource library containing the evidence and tools used to develop the curriculum, along with additional resources aligned to each module. The full curriculum and supporting materials are publicly available through the Climate Advocacy Lab website (see Footnote 1).

**Table 1:** Curriculum overview for the climate and health PLC.

Component	Description
Curriculum focus	Practice-based climate advocacy and communication; evidence-informed message strategy; narrative and storytelling for engagement; relational organizing and peer learning; applied campaign and systems strategy
Learning objectives	Participants will be able to: Explain the connections between climate change and human health using a planetary health framework Apply evidence-based advocacy frameworks to develop climate and health campaign strategies Identify key stakeholders and mobilize professional and community networks to advance climate-health initiatives Develop audience-centered messages and strategic communication plans for climate and health advocacy Use storytelling and narrative strategies to communicate climate and health risks and solutions Identify strategies to sustain and evaluate climate action through partnerships and coalition building
Program structure	In-person modules: Climate and Health Foundations; Campaign Strategy and Development; Community Organizing and Network Mobilization; Strategic Messaging and Communication; Narrative Development and Storytelling; Personal Climate Narrative; Climate Communication for Public Engagement; Sustaining Advocacy and Engagement Virtual modules: Strategic Planning for Advocacy; Digital Advocacy and Storytelling; Coalition Building and Movement Sustainability
Evaluation	Baseline intake survey; post-session surveys; end-of-program evaluation; follow-up surveys (3 and 6 months)

## Outcomes

*Four regional Climate and Health Peer Learning Circle cohorts were implemented in 2025:*

- Midwest Cohort (March): 14 participants
- Northeast Cohort (April–May): 11 participants
- West Cohort (Sept–Oct): 16 participants
- South Cohort (Oct–Nov): 11 participants

In total, 52 health professionals completed the program in 2025. The PLC also convened its first cross-cohort alumni gathering, bringing together participants from the virtual pilot cohorts and the Midwest and Northeast regional cohorts. Activities included participant spotlights, campaign strategy workshops, peer learning, and cross-regional networking. A national summit is being explored for 2026 to further support collaboration, peer-learning, and community building.

### Practice-based outcomes

To understand how learning translated into practice over time, the PLC incorporated structured feedback and reflection throughout the program lifecycle. Participants completed surveys prior to the in-person training and immediately following the 2-day workshop, and at 3 and 6 months after the month-long intensive. These touchpoints were designed to capture shifts in confidence, skill application, and sustained engagement, rather than to assess causal outcomes.

In addition, participant reflections were gathered through structured prompts, facilitated discussions, and coaching sessions. Together, these data were treated as practice-based evidence, offering insight into how leadership skills were applied in real-world contexts and how momentum was sustained beyond the formal training period.

Pre-training responses indicated that participants entered the program with a strong interest in strengthening communication skills, expanding collaborative networks, and developing practical strategies for climate and health advocacy. Participants also described several barriers to climate-health engagement, including difficulty translating complex scientific evidence into accessible messages, limited institutional support for advocacy activities, and challenges engaging diverse audiences across political and community contexts.

Survey responses from participants across the four regional cohorts suggest increases in confidence related to several core advocacy competencies. Pre- and post-training responses indicated consistent gains in participants’ self-reported confidence in developing climate and health messages, understanding the connections between climate change and health, relational organizing with non-health professionals, and campaign planning. The largest increases were observed in understanding climate-health connections and in narrative development for advocacy, with post-training confidence scores approaching the upper range of the 5-point confidence scale across cohorts. Additional evaluation results and survey instruments are provided in Supplementary material.

Participant reflections further highlighted the value of peer learning, storytelling, and the in-person training environment. Many respondents described the opportunity to connect with other health professionals working in climate and health as particularly meaningful, noting that the cohort structure supported relationship-building, shared learning, and motivation to continue advocacy efforts.

Additional evaluation results, survey instruments, and participant reflections are provided in Supplemental material.

### Emerging impact

Although the PLC was not designed as a research study, participant reflections provide insight into how training translated into practice. Alumni reported applying PLC messaging strategies to tailor communication to specific audiences, frame climate as an urgent health issue, and link individual experiences to broader structural and policy determinants. Several described using familiar health language, such as risk, resilience, and safety, to help their audience recognize climate change as a core health issue. These observations align with evidence that health professionals communicate most effectively when messaging reflects audience priorities (3, 34).

Participants also described combining data and personal narratives to make climate-related health issues more actionable. For example, community health professionals designed outreach initiatives that integrated health data with lived experience, which they perceived as increasing community engagement. Evidence suggests that pairing stories with data strengthens public and policymaker engagement (35, 36).

Some participants applied PLC tools, such as system mapping, narrative framing, and structured peer-problem-solving, to strengthen community-level environmental and health projects. They reported greater clarity in project goals, improved team coordination, and stronger alignment with community priorities. These experiences reflect the importance of goal clarity and collaborative processes in community initiatives (37).

At the national level, alumni described using program skills to assume leadership roles in climate-health and environmental justice initiatives, contribute to curriculum and resource development, mobilize professional networks, and deliver educational presentations. Together, these examples illustrate how practice-based, peer-informed leadership development can extend the reach of health professionals working at the intersection of climate and health (5, 32).

## Discussion

The PLC demonstrates what becomes possible when health professionals are supported to move beyond knowledge acquisition to practice-based leadership. Participants applied skills directly to active projects and campaigns, testing messages, strategies, and approaches in real-world settings while learning with peers navigating similar constraints. This environment supported the development of communication and advocacy approaches that were both credible and context specific (7, 38). While professional trust provided an important foundation, sustained engagement emerged through repeated application, peer discourse, and shared accountability.

The program also surfaced tensions common in climate and health work. One involved balancing advocacy and expectations of professional neutrality (7, 13). Participants described working within institutional cultures that discourage public engagement, even as climate-related health impacts become increasingly visible. These constraints were often shaped by political polarization, organizational risk management, or formal limits on policy engagement (10, 11). Rather than ignoring these realities, the PLC helped participants in developing strategies—such as health-centered framing, values-based communication, and audience awareness—to advance action while navigating professional boundaries.

A second tension emerged between institutional constraints and community accountability. Participants based in health systems, academic institutions, or government settings often faced limits on messaging, partnerships, while those in community or advocacy organizations reported greater flexibility but fewer resources and less institutional protection (6, 38). The PLC’s cross-sector design created space for these perspectives to inform one another, enabling participants to adapt strategies across contexts while remaining accountable to community needs.

Several limitations warrant consideration. Participation in the PLC is voluntary, and participants may already be more inclined toward advocacy than the broader health workforce. The program also requires a substantial time commitment, which may pose barriers for individuals with heavy workloads, caregiving responsibilities, or limited employer support. Political and organizational constraints further shape how learning can be applied (10, 13). Although the PLC centers equity and justice, continued effort is needed to reduce participation barriers and expand access for those most affected by climate-related health inequities.

Despite these challenges, the PLC offers important insights for climate and health education. In politically complex and constrained environments, training models that emphasize adaptability, narrative skill, relational organizing, and peer support may be particularly valuable (11, 38). Rather than relying solely on information dissemination or individual behavior change, the PLC demonstrates the value of investing in collective leadership capacity and peer networks that help health professionals stay engaged over time (26, 38).

Collaborative scholarship also emerged from this approach. Co-authoring this manuscript with a PLC alum reflects the program’s emphasis on practitioner-led knowledge creation and distributed leadership, modeling the same peer-to-peer learning and shared accountability the PLC is designed to foster. This challenges traditional hierarchies of expertise and underscores the value of lived experience and applied practice in advancing climate and health scholarship.

Sustaining momentum beyond the training itself requires ongoing infrastructure. The PLC’s community of practice provides a mechanism for continued connection, shared learning, and collaboration (18). Expanding cross-sector partnerships and strengthening these networks may further amplify impact and support collective problem-solving.

### Implications for health education and promotion

The PLC suggests several implications for the future of climate and health education and promotion. First, programs should move away from one-time, content-heavy training and toward models that emphasize application, reflection, and peer learning over time (6, 19). Sustained engagement is especially important in political and institutional contexts where conversations about climate and health are constrained or contested (6, 7, 23).

Second, health education must prepare learners for the realities of practice. This includes developing skills in strategic framing, audience awareness, and relationship-building, alongside an understanding of how to advance change within professional, legal, and organizational boundaries (2, 13). Working effectively under constraint is now central to climate and health leadership.

Third, institutions and funders play a critical role. Investments in peer networks, coaching, and communities of practice can help translate individual learning into collective impact, particularly when formal policy pathways are limited (6, 11). Funding structures that support flexibility, cross-sector collaboration, and authentic community partnership are essential for sustaining momentum.

Finally, the PLC suggests that health education and promotion should place less emphasis on neutrality and information delivery alone, and greater emphasis on building the skills, relationships, and confidence required for civic engagement (12, 22). In a moment of accelerating climate-related health impacts, equipping health professionals to act as trusted, adaptive leaders is a core component of effective health promotion (11).

## Conclusion

The PLC demonstrates that peer-to-peer learning is an effective model for cultivating climate-focused leadership across diverse health disciplines. Through a structured curriculum that integrates real-time application, practical skill-building, narrative strategy, systems thinking, and opportunities for collective action, participants move beyond acquiring knowledge to practicing leadership in a supportive, collaborative environment (5, 23, 27, 28). Graduates have applied their learning to strengthen communication, lead community initiatives, and assume leadership roles at local and national levels. Co-authoring this manuscript with a PLC alum embodies the spirit of the program: shared leadership, mutual accountability, and community-rooted expertise.

Beyond climate-focused work, this approach offers a transferable model of leadership development that can be adapted to strengthen health professional engagement across other social justice and public interest issues. Health professionals remain among the most trusted messengers in society, and structured peer-learning models like the PLC can help translate that trust into sustained leadership and collective action (23, 25).

Looking ahead, this model points toward the value of cross-sector, community-embedded leadership development. Expanding partnerships with public policy, urban planning, environmental justice, and community organizing professionals may broaden the relevance and impact of climate and health advocacy. By integrating health into climate work and strengthening networks across sectors, the PLC supports systemic change and helps build healthier, more resilient communities.

## Data Availability

The original contributions presented in the study are included in the article/Supplementary material, further inquiries can be directed to the corresponding author.

## Footnotes

1 Climate Advocacy Lab. Climate and Health Peer Learning Circle. Available at: https://climateadvocacylab.org/climate-health-peer-learning-circle-2025
