# Supplementary material for: From knowledge to collective action: a peer-led model for climate and health leadership
Source: Front Public Health. 2026 Mar 30;14:1805050. doi: 10.3389/fpubh.2026.1805050 (PMC13070829; doi:10.3389/fpubh.2026.1805050)
Supplement: Supplementary file 1 [file Data_Sheet_1.pdf]

What is the Climate + Health PLC?

The Climate + Health Peer Learning Circle is a *free training program* designed *for experienced leaders at the intersection of climate and health*. This regional initiative combines in-person workshops and virtual sessions to foster peer learning and collaboration.

Participants will refine their leadership skills, gain strategies for community engagement, and exchange insights with a network of accomplished peers.

What The Circle Is vs. What It Is Not

- Collaborative and Peer-Informed
- A space for “I don’t know”
- Responsive
- Relationship-driven
- Take what you need, leave the rest

- A one-way street
- Having all the answers
- Set in stone
- Siloed

Goal:

To empower health professionals working at the intersection of climate and health with the skills, tools, and collaborative networks needed to communicate effectively, drive advocacy, and implement evidence-based solutions that inspire meaningful climate action within their communities and professional networks

Core Competencies

Climate Advocacy & Communication – Strengthen your climate advocacy and communication skills by learning evidence-based strategies, including how to share your climate story, build personal influence, and reshape climate narratives.

Community Engagement & Activation – Build skills and confidence to effectively use your own communication channels and engage your community in climate action.

Sustained Climate Action – Gain practical techniques for inspiring, maintaining, and measuring climate action within your community or audience.

Peer Learning & Collaboration – Cultivate supportive relationships within a network of peer experts working at the intersection of climate and health, sharing knowledge and strategies to overcome common challenges.

The C+H Circle Change Model

Train health professionals at the intersection of climate and health with evidence-based communication strategies and tools

Use a train-the-trainer model to expand climate engagement within networks

Strengthen organizations by bringing back proven practices and mobilizing trusted health messengers

Build a growing community of practice to sustain and grow climate leadership nationwide
