# Supplementary material for: From knowledge to collective action: a peer-led model for climate and health leadership
Source: Front Public Health. 2026 Mar 30;14:1805050. doi: 10.3389/fpubh.2026.1805050 (PMC13070829; doi:10.3389/fpubh.2026.1805050)
Supplement: Supplementary file 2 [file Data_Sheet_2.pdf]

Climate and Health Peer Learning Circle Evaluation Summary (2025)

Evaluation Overview

Evaluation data were collected from participants in the Climate and Health Peer Learning Circle (PLC) across four regional cohorts (Midwest, Northeast, Southern, and Western) during 2025. Data were gathered through voluntary pre-training and post-training surveys administered before the in-person kickoff training and immediately following the two-day workshop. Additional reflections were collected through structured prompts during program activities.

Surveys included Likert-scale questions assessing participant confidence in key advocacy competencies as well as open-ended questions regarding learning goals, barriers to climate-health advocacy, and perceived value of the training. Results were summarized descriptively to identify patterns in participant responses. These findings are presented as **practice-based evaluation results rather than formal research outcomes**.

Participant Demographics

A total of **52 participants** took part in the Climate and Health Peer Learning Circle across multiple U.S. regions.

Participants represented multiple professional sectors including academia, healthcare and clinical practice, public health and government, and nonprofit advocacy organizations.

Professional Roles Represented

Academic and Research Roles
Participants included assistant professors, postdoctoral scholars, research scientists, research and evaluation specialists, and program directors working in universities and research institutions.

Public Health and Government Roles
Participants included professionals working in public health nursing, environmental justice offices, city and state health departments, and tribal health organizations.

Non-Profit and Advocacy Roles
Participants represented environmental, climate justice, and health advocacy organizations working at local, regional, and national levels.

Clinical and Direct Care Roles
Participants included registered nurses, family medicine physicians, doulas, and clinical therapists.

Pre-Training Survey Findings

Barriers to Climate and Health Advocacy

Participants described several barriers to engaging in climate and health advocacy.

Knowledge and Data Gaps

Participants reported difficulty accessing clear and trustworthy data, identifying effective interventions, and benchmarking progress in climate-health initiatives.

Public Awareness and Engagement

Respondents noted that many audiences are overwhelmed, skeptical, or disengaged from climate discussions. Misinformation and difficulty communicating the connections between climate change and human health were commonly cited challenges.

Political and Institutional Constraints

Participants described barriers including limited political will, slow institutional change processes, and challenges advocating in politically polarized contexts.

Resource and Capacity Limitations

Limited funding, lack of institutional support, and competing professional responsibilities were frequently reported barriers.

Advocacy Strategy and Communication

Participants expressed uncertainty about planning effective campaigns, engaging legislators, and translating scientific evidence into messages accessible to diverse audiences.

Example participant reflections:

“Consistent messaging across all community members, including small rural communities, tribal communities, and various advocacy groups. The level of understanding varies so much and it's hard to get everyone on the same page while also being respectful of tribal sovereignty and knowledge.”

“Making the connection of a complex topic. A lot of the solutions for climate are larger and structural including capitalism and colonialism, so sometimes the issues seem very large and difficult to address and communicate.”

Participant Learning Goals

Participants entered the training with several learning goals related to strengthening their effectiveness as climate and health advocates.

Communication and Framing Skills

Many participants aimed to improve their ability to translate scientific information into accessible language and craft messages that resonate with policymakers, communities, and cross-sector partners.

Networking and Collaboration

Participants sought to build strategic partnerships, expand professional networks, and collaborate with peers across sectors.

Leadership and Campaign Development

Participants described goals related to strengthening advocacy leadership, organizing campaigns, and mobilizing colleagues and community members.

Knowledge and Resource Development

Participants expressed interest in identifying evidence-based advocacy strategies, tools, and funding opportunities to support climate-health initiatives.

Applying an Equity Lens

Respondents emphasized the importance of integrating racial and environmental justice considerations into climate and health advocacy.

Example participant reflections:

"Through the Circle, I aim to strengthen my ability to translate scientific evidence into accessible, equity-centered policy communication."

"Better understanding of adequately communicating the interconnectedness of climate and health to healthcare and non-healthcare professionals."

Post-Training Survey Findings

Skills Participants Reported Increased Confidence In

Storytelling and Narrative Development

Participants reported increased confidence in developing and sharing personal climate-health stories and using narrative as a communication strategy.

Audience Engagement and Message Development

Participants described greater confidence in identifying target audiences and tailoring messages based on audience values and perspectives.

Campaign Strategy and Advocacy

Respondents reported gaining skills in power mapping, advocacy strategy development, and understanding the long-term structure of campaigns.

Communication Tools and Digital Engagement

Participants indicated increased confidence in communication strategies, including social media engagement and digital advocacy tools.

Example reflections:

“I feel most confident in the value and practice of storytelling as well as in my ability to name and choose different advocacy strategies.”

“Using concepts of climate storytelling and theory of change to build campaigns with evidence-based advocacy.”

Perceived Value of the Training

Participants highlighted several aspects of the PLC experience as particularly valuable.

Peer Learning and Community Building

Participants emphasized the importance of connecting with other health professionals working on climate and health initiatives.

Narrative and Storytelling

Narrative development and personal storytelling exercises were frequently described as meaningful components of the training.

In-Person Learning Environment

Participants noted that the in-person format supported deeper dialogue, relationship building, and collaborative problem solving.

Relational Organizing and Advocacy Strategy

Participants valued learning the Climate Advocacy Lab’s approach to relational organizing and advocacy strategy.

Example reflection:

“The most valuable aspects for me were opportunities for open dialogue, knowledge sharing, and the processing of grief with my cohort.”

Areas Where Participants Requested Additional Support

Participants identified several areas where additional support would be helpful.

These included:

• campaign implementation and strategy development
• communication and messaging development
• engaging policymakers and decision makers
• funding and program sustainability
• opportunities for continued peer coaching and feedback

Pre-Post Training Confidence Assessment

Survey Response Rates

Pre-training responses: **41 participants**
Post-training responses: **34 participants**

Confidence Scale

Participants rated their confidence using a five-point Likert scale:

1 – Not confident
2 – Slightly confident
3 – Somewhat confident
4 – Confident
5 – Very confident

Summary of Findings

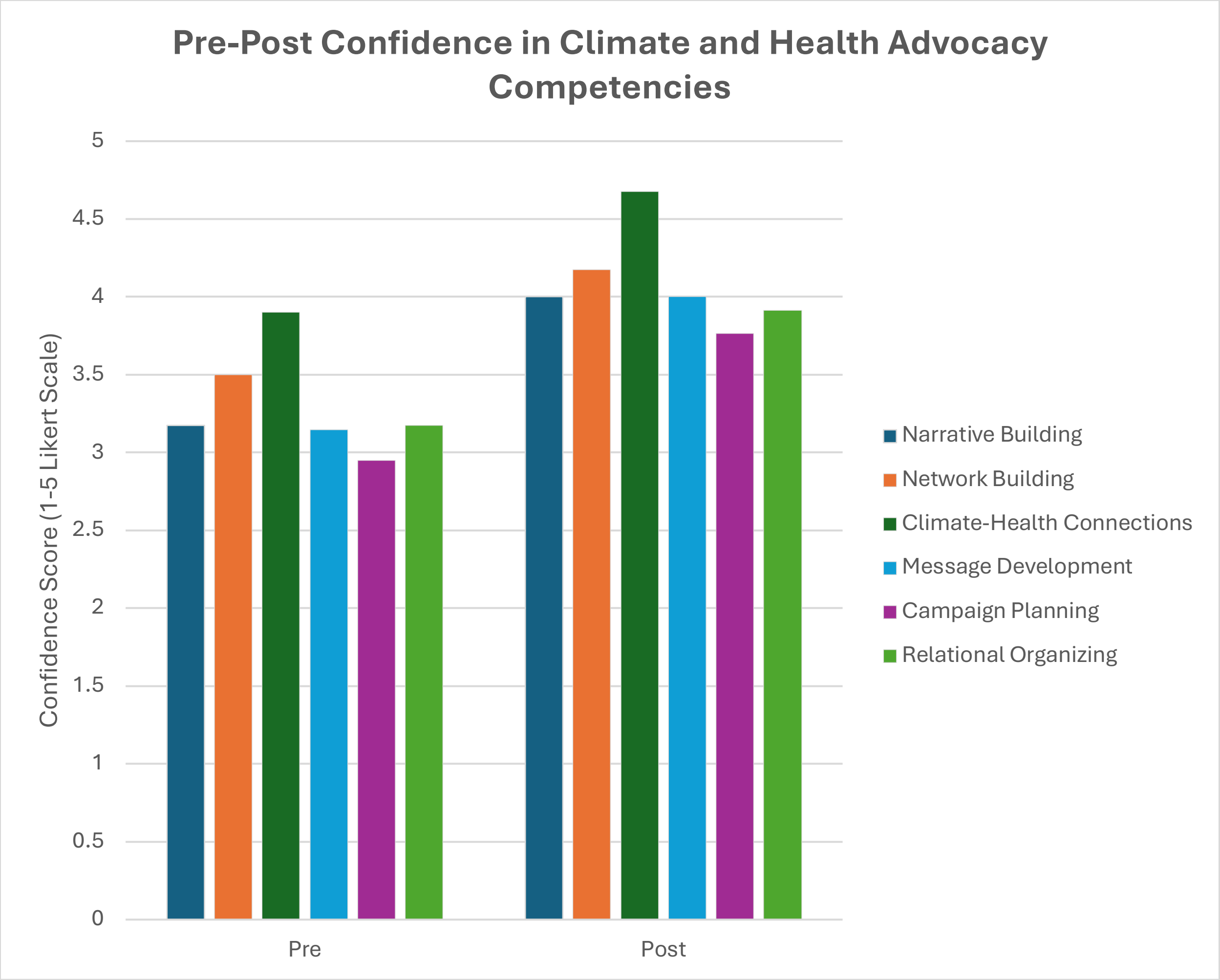

Across all four regional cohorts, participants demonstrated consistent increases in confidence across all competency areas from pre-training to post-training.

The largest increases were observed in:

• understanding the connections between climate change and health
• narrative development and storytelling for advocacy
• developing and delivering climate-health messages
• campaign planning and advocacy strategy
• network building and coalition engagement

Post-training confidence scores across most competencies approached 4.5–5 on the five-point scale, indicating high levels of participant confidence following the training.

Due to limited post-training responses from the Southern cohort (n=3), regional comparisons should be interpreted cautiously.

Overall, these findings suggest that the PLC training strengthened participants' readiness and confidence to engage in climate and health advocacy across diverse professional contexts.
